# Supplementary material for: Tumor microenvironment governs the prognostic landscape of immunotherapy for head and neck squamous cell carcinoma: A computational model-guided analysis
Source: PLoS Comput Biol. 2025 Jun 3;21(6):e1013127. doi: 10.1371/journal.pcbi.1013127 (PMC12162103; doi:10.1371/journal.pcbi.1013127)
Supplement: S1 Text — (PDF) [file pcbi.1013127.s001.pdf]

**S1 Text: Mathematical representation of the fluxes corresponding to the HNSCC TME network**

| Reaction flux | Mathematical expression |
|---|---|
| Proliferation of CST ($F_{ResCST}$) | $ResK_{RCST}CST\left(1-\frac{CST}{(1-I_a)Y_{CST}+1}\right)\alpha_C$<br>$\alpha_C := \frac{1}{a_{comp}(CPDL1+CNPDL1)+1}$<br>$I := \tanh(\alpha K_{CAFB}CAF)$<br>$\alpha$: Proportion of CAF engaged in barrier formation<br>$K_{CAFB}$: Barrier formation rate |
| Exhausted T-cells-driven proliferation modulator of tumor cells ($F_{TEXC}$) | $\left(1+K_{TXC}\frac{TEX}{TEX+1}\right)$ |
| CAF-driven proliferation modulator of T-exposed Tumor cells ($F_{CAFC}$) | $\left(1+K_{CAFC}\frac{CAF}{CAF+1}\right)$ |
| Conversion from CST to CNPDL1($F_{CSTCNPDL1}$) | $K_{CSTCNPDL1}\left(\frac{IL8}{IL8+1}\right)CST$ |
| Conversion from CST to CPDL1($F_{CSTCPDL1}$) | $K_{CSTCPDL1}\left(\frac{IL8}{IL8+1}\right)CST$ |
| Killer T cell-driven elimination($F_{TKCST}$) | $K_{TKC}CST(TKPD1+TKNPD1)\gamma\beta$<br>$\gamma := \frac{IFNG}{IFNG+1}$<br>$\beta := \frac{1}{K_{LAC}LAC+1}$ |
| Death of CST ($F_{DCST}$) | $(K_{CSTD}+\delta_{IFNGCSTD}+\delta_{M1CSTD})CST$<br>$\delta_{IFNGCSTD} := K_{IFNGCSTD}\frac{IFNG}{IFNG+1}$<br>$\delta_{M1CSTD} := K_{M1CSTD}\frac{MACM1}{MACM1+1}$ |

| | |
|---|---|
| Proliferation of CSNT ($F_{RESCSNT}$) | $ResK_{RCSNT}CSNT\left(1-\frac{CSNT}{I_aY_{CST}+1}\right)\alpha_{C1}$ <br> $\alpha_{C1} \coloneqq \frac{1}{\alpha_{comp}(CRNPDL1+CRPDL1)+1}$ |
| Exhausted T cell-driven proliferation for immune inaccessible tumor cells ($F_{TEXCR}$) | $\left(1+\alpha_T K_{TXC}\frac{TEX}{TEX+1}\right)$ <br> $\alpha_T \coloneqq \exp(-\delta\alpha^2 CAF^2)$ <br> $\delta$: Width of CAF barrier, $\alpha$: proportion of CAF engaged in barrier forming |
| CAF-driven proliferation for immune inaccessible tumor cells ($F_{CAFCR}$) | $\left(1+K_{CAFCR}\frac{CAF}{CAF+1}\right)$ |
| Conversion from immune inaccessible stem to immune inaccessible tumor cells ($F_{CSNTCRNPDL1}$) | $K_{CSNTCNPDL1}CSNT\frac{IL8}{IL8+1}$ |
| Conversion from immune inaccessible stem to immune inaccessible PDL1+ tumor cells ($F_{CSNTCRPDL1}$) | $K_{CSNTCPDL1}CSNT\frac{IL8}{IL8+1}$ |
| Killer T cell-driven elimination of immune inaccessible tumor stem cell ($F_{TKCSNT}$) | $K_{TKC}\alpha_T(TKPD1+TKNPD1)CSNT\gamma\beta$ |
| Death of immune-inaccessible stem cells ($F_{DCSNT}$) | $F_{CSTD}\frac{CSNT}{CST}$ |
| Resource-driven growth of PDL1-immune-accessible tumor cells ($F_{RESCNPDL1}$) | $ResK_{RCNPDL1}\gamma_{CNPDL1}CNPDL1\alpha_{C2}$ <br> $\alpha_{C2} = \frac{1}{\alpha_{Comp}(CST+CPDL1)+1}$ <br> $\gamma_{CNPDL1} = \left(1-\frac{CNPDL1}{(1-I_a)Y_{CNPDL1}+1}\right)$ |

| | |
|---|---|
| Killer T cell-driven elimination of PDL1- immune-accessible tumor cells ($F_{TKCNPDL1}$) | $K_{TKCNPDL1}CNPDL1(TKPD1+TKNPD1)\gamma\beta$ |
| Conversion from PDL1- to PDL1+ tumor cells ($F_{CNPDL1CPDL1}$) | $K_{CPDNPD}\frac{IFNG}{IFNG+1}CNPDL1$ |
| Death of PDL1-, immune-accessible tumor cells ($F_{DCNPDL1}$) | $K_{CNPDL1D}CNPDL1$ |
| Resource-driven growth of PDL1+, immune-accessible tumor cells ($F_{RESCPDL1}$) | $ResK_{RCPDL1}\gamma_{CPDL1}CPDL1\alpha_{C3}$<br>$\alpha_{C3}=\frac{1}{\alpha_{Comp}(CST+CNPDL1)+1}$<br>$\gamma_{CPDL1}=\left(1-\frac{CPDL1}{(1-I_a)Y_{CPDL1}+1}\right)$ |
| Killer T cell-driven elimination of PDL1+ immune-accessible tumor cells($F_{TKNPDCPDL1}$) | $K_{TKCPDL1}CPDL1(TKNPD1)\gamma\beta$ |
| Death of PDL1+, immune-accessible tumor cells ($F_{DCPDL1}$) | $K_{CPDL1D}CPDL1$ |
| Resource-driven growth of PDL1-, immune-inaccessible tumor cells ($F_{ResCRNPDL1}$) | $ResK_{RCRNPDL1}\gamma_{CRNPDL1}CRNPDL1\alpha_{C4}$<br>$\alpha_{C4}=\frac{1}{\alpha_{Comp}(CSNT+CRPDL1)+1}$<br>$\gamma_{CRNPDL1}=\left(1-\frac{CRNPDL1}{I_aY_{CRNPDL1}+1}\right)$ |
| Death of PDL1-, immune-inaccessible tumor cells ($F_{DCRNPDL1}$) | $K_{CNPDL1D}CRNPDL1$ |
| Killer T cell-driven elimination of immune-inaccessible, PDL1- tumor cells ($F_{TKCRNPDL1}$) | $K_{TKC}CRNPDL1T_K\gamma\beta\alpha_T$<br>$T_K\coloneqq(TKPD1+TKNPD1)$ |

| | |
|---|---|
| IFNG-induced conversion to PDL1+ immune-inaccessible Tumor cells ($F_{CRNPDL1CRPDL1}$) | $K_{CPDNPD}\frac{IFNG}{IFNG+1}CRNPDL1$ |
| Resource-driven growth of PDL1+, immune-inaccessible tumor cells ($F_{ResCRPDL1}$) | $ResK_{RCRPDL1}\gamma_{CRPDL1}CRPDL1\alpha_{C5}$<br>$\alpha_{C5}=\frac{1}{\alpha_{Comp}(CSNT+CRNPDL1)+1}$<br>$\gamma_{CRNPDL1}=\left(1-\frac{CRPDL1}{I_a Y_{CRPDL1}+1}\right)$ |
| Death of PDL1+, immune-inaccessible tumor cells ($F_{DCRPDL1}$) | $K_{CPDL1D}CRPDL1$ |
| Proliferation of Killer PD1+ T cells ($F_{\mathrm{Pro}\,TKPD1}$) | $K_{TKPD}TKPD1\left(1-\frac{TKPD1}{Y_{TKM}-TEX-TKNPD1+1}\right)$ |
| M1 macrophage, Helper-driven growth of Killer T cells ($F_{THTKPD1}$) | $1+K_{THTK}\frac{TH\ MACM1}{MACM1\ TH+1}$ |
| IL2-driven growth of Killer T cells ($F_{IL2TK}$) | $1+K_{IL2TK}\frac{IL2}{IL2+1}$ |
| Effect of anti-PD1(u): Conversion from PD1+ to PD1- killer T cell($F_{TKPD1TKNPD1}$) | $K_{TKPDNPD1}TKPD1u$<br>$u$: Anti-PD1 dosage |
| Exhaustion rate ($F_{TKPD1TEX}$) | $K_{TKPDTEX}T_{KPD1}\frac{CPDL1\ MACM2}{CPDL1\ MACM2+1}$ |
| Death rate of PD1+ killer T cell ($F_{DTKPD1}$) | $K_{TKPDD}TKPD1$ |
| Proliferation of PD1- Killer T cells($F_{\mathrm{Pro}\,TKNPD1}$) | $K_{TKNPD}TKNPD1\left(1-\frac{TKNPD1}{Y_{TKM}-TEX-TKPD1+1}\right)$ |

| | |
|---|---|
| Helper-driven growth of Killer T cells ($F_{THTKNPD1}$) | $1 + uK_{THTK}\frac{TH}{TH+1}$ |
| Death rate of PD1-Killer T cells ($F_{DTKNPD1}$) | $K_{TKPDD}TKNPD1$ |
| Proliferation rate of helper T cells ($F_{\mathrm{Pro}\,TH}$) | $K_{TH}\left(1 - \frac{TH}{Y_{TH}}\right)$ |
| Growth via antigen sensing ($F_{CANTH}$) | $1 + K_{CANTH}\frac{(CNPDL1 + uCPDL1)}{(CNPDL1 + uCPDL1 + 1)}$ |
| Regulator-driven inhibition ($F_{TREGTH}$) | $\frac{1}{1 + K_{REGTH}TREG}$ |
| Death of helper T cells ($F_{DTH}$) | $K_{THD}TH$ |
| Proliferation of regulatory T cells ($F_{\mathrm{Pro}\,TREG}$) | $K_{TREG}TREG\left(1 - \frac{TREG}{Y_{TREG}}\right)$ |
| CAF-driven proliferation of Regulatory T cells ($F_{CAFTREG}$) | $1 + K_{CAFTREG}\frac{CAF}{CAF+1}$ |
| Death rate of Regulatory T cells ($F_{DTREG}$) | $K_{TREGD}TREG$ |
| Proliferation of exhausted T cells ($F_{\mathrm{Pro}\,TEX}$) | $K_{TEX}TEX\left(1 - \frac{TEX}{Y_{TKM} - TKNPD1 - TKPD1 + 1}\right)$ |
| Death rate of exhausted T cells ($F_{DTEX}$) | $K_{TEXD}TEX$ |
| Proliferation of wild-type fibroblasts ($F_{\mathrm{Pro}\,FWT}$) | $K_{FWT}FWT\left(1 - \frac{FWT}{Y_{FM} - CAF + 1}\right)$ |

| | |
|---|---|
| Conversion from wild type to invasive fibroblasts ($F_{FWTCAF}$) | $K_{FWTCAF}\frac{(\alpha_{LIFFWT}\,LIF)^2}{(\alpha_{LIFFWT}\,LIF)^2+K_{LIFT}}FWT$<br>$\alpha_{LIFFWT}$: Proportion of LIF in contact with FWT |
| Death of wild type fibroblasts ($F_{DFWT}$) | $K_{FWTD}FWT$ |
| Proliferation of invasive fibroblasts ($F_{\mathrm{Pr}oCAF}$) | $K_{CAF}CAF\left(1-\frac{CAF}{Y_{FM}-FWT+1}\right)$ |
| OPN-induced growth of invasive fibroblasts ($F_{OPNCAF}$) | $1+K_{OPNCAF}\frac{OPN}{OPN+1}$ |
| M2 macrophage-induced growth ($F_{M2CAF}$) | $1+K_{M2CAF}\frac{MACM2}{MACM2+1}$ |
| Tumor cells-driven growth ($F_{CANCAF}$) | $1+K_{CTCAF}\left(\frac{TUM_{AC}}{TUM_{AC}+1}+K_{CTCAFR}\frac{TUM_{IAC}}{TUM_{IAC}+1}\right)$<br>$TUM_{AC} \coloneqq (CST+CPDL1+CNPDL1)$<br>$TUM_{IAC} \coloneqq (CSNT+CRPDL1+CRNPDL1)$ |
| Conversion from invasive to wild type fibroblasts ($F_{CAFFWT}$) | $K_{CAFFWT}CAF$ |
| Death of invasive fibroblasts ($F_{DCAF}$) | $K_{CAFD}CAF$ |
| Proliferation of M1-macrophages ($F_{\mathrm{Pr}oMACM1}$) | $K_{M1}MACM1\left(1-\frac{MACM1}{Y_{MM}-MACM2}\right)$ |
| Proliferation of M1 macrophage via antigen-sensing ($F_{CANMACM1}$) | $1+K_{CANM1}\frac{(TUM_{PDL1-}+uTUM_{PDL1+})}{(TUM_{PDL1-}+uTUM_{PDL1+}+1)}$<br>$TUM_{PDL1-} \coloneqq (CST+CSNT+CNPDL1+CRNPDL1)$<br>$TUM_{PDL1+} \coloneqq (CPDL1+CRPDL1)$ |
| Conversion from M1 to M2 macrophage ($F_{M1M2}$) | $K_{M1M2}\frac{(\alpha_{IL10M1}IL10)^2}{(\alpha_{IL10M1}IL10)^2+1}$<br>$\alpha_{IL10M1} \coloneqq$ Proportion of IL-10 in contact with MACM1 |

| | |
|---|---|
| Conversion from M2 to M1 macrophage ($F_{M2M1}$) | $K_{M2M1}MACM2$ |
| Death of M1 macrophage ($F_{DMACM1}$) | $K_{M1D}MACM1$ |
| Proliferation of M2 macrophage ($F_{\Pr oMACM2}$) | $K_{M2}MACM2\left(1-\frac{MACM2}{Y_{MM}-MACM1+1}\right)$ |
| CAF-driven growth of M2 macrophage ($F_{CAFMACM2}$) | $K_{CAFM2}\frac{CAF}{CAF+1}$ |
| Death of M2 macrophage ($F_{DMACM2}$) | $K_{M2D}MACM2$ |
| IL-2 secretion by Killer Cells ($F_{TKIL2}$) | $K_{TKIL2}(TKPD1+TKNPD1)$ |
| Degradation of IL-2 ($F_{DIL2}$) | $K_{IL2D}IL2$ |
| LIF secretion by CAF ($F_{CAFLIF}$) | $K_{CAFLIF}CAF$ |
| LIF secretion by Tumor cells ($F_{CANLIF}$) | $K_{CANLIF}(TUM_{AC}+TUM_{IAC})$ |
| Degradation of LIF ($F_{DLIF}$) | $K_{LIFD}LIF$ |
| IFNG secretion by T cells ($F_{TKIFNG}$) | $K_{TIFNG}(TKPD1+TKNPD1)$ |
| Inhibition of IFNG secretion by OPN ($F_{OPNIFNG}$) | $\frac{1}{\alpha_{OPNIFNG}OPN+1}$ <br> $\alpha_{OPNIFNG}$: Proportion of OPN in contact with IFNG |

| | |
|---|---|
| Degradation of IFNG ($F_{DIFNG}$) | $K_{IFNGD}IFNG$ |
| IL-8 secretion by M2 macrophage ($F_{M2IL8}$) | $K_{M2IL8}MACM2$ |
| IL8-secretion by CAF ($F_{CAFIL8}$) | $K_{CAFIL8}CAF$ |
| IL8-secretion by tumor cells ($F_{CAFIL8}$) | $K_{CAFIL8}(TUM_{AC} + TUM_{IAC})$ |
| Degradation of IL8 ($F_{DIL8}$) | $K_{IL8D}IL8$ |
| Lactate secretion by tumor cells ($F_{CANLAC}$) | $K_{CANLAC}(TUM_{AC} + TUM_{IAC})$ |
| Lactate secretion by M2 macrophage ($F_{M2LAC}$) | $K_{M2LAC}MACM2$ |
| Lactate degradation ($F_{DLAC}$) | $K_{LACD}LAC$ |
| IL10 secretion by Killer T cells ($F_{TKIL10}$) | $K_{TKIL10}(TKPD1 + TKNPD1)$ |
| Degradation of IL10 ($F_{DIL10}$) | $K_{IL10D}IL10$ |
| ICAM1 secretion by Killer T cells ($F_{TKICAM1}$) | $K_{TKICAM}(TKPD1 + TKNPD1)$ |
| ICAM1 degradation ($F_{DICAM1}$) | $K_{ICAM1}ICAM1$ |
| OPN secretion by CAF ($F_{CAFOPN}$) | $K_{CAFOPN}CAF$ |
| OPN secretion by tumor cells ($F_{CANOPN}$) | $K_{CANOPN}(TUM_{AC} + TUM_{IAC})$ |

| | |
|---|---|
| Inhibition of OPN secretion by IRF8 ($F_{IRF8OPN}$) | $$\frac{1}{\alpha_{IRF8OPN} IRF8 + 1}$$ $\alpha_{IRF8OPN}$: Proportion of IRF8 in contact with OPN |
| Degradation of OPN ($F_{DOPN}$) | $K_{OPND} OPN$ |
| IRF8 secretion by M1 macrophage ($F_{M1IRF8}$) | $K_{M1IRF8} MACM1$ |
| Degradation of IRF8 ($F_{DIRF8}$) | $K_{IRF8D} IRF8$ |
