# Supplementary material for: Tumor microenvironment governs the prognostic landscape of immunotherapy for head and neck squamous cell carcinoma: A computational model-guided analysis
Source: PLoS Comput Biol. 2025 Jun 3;21(6):e1013127. doi: 10.1371/journal.pcbi.1013127 (PMC12162103; doi:10.1371/journal.pcbi.1013127)
Supplement: S2 Text — (PDF) [file pcbi.1013127.s002.pdf]

S2 Text: Overall mathematical model

Given the fluxes in Supplementary Table 1, we construct the overall mathematical model for the HNSCC TME as:

$$\frac{dCST}{dt} = F_{RESCST}F_{TEXC}F_{CAFC} - F_{CSTCNPDL1} - F_{CSTCPDL1} - F_{TKCST} - F_{DCST}$$

$$\frac{dCSNT}{dt} = F_{RESCSNT}F_{TEXCR}F_{CAFCR} - F_{CSNTCRNPDL1} - F_{CSNTCRPDL1} - F_{TKCSNT} - F_{DCSNT}$$

$$\frac{dCNPDL1}{dt} = F_{RESCNPDL1}F_{TEXC}F_{CAFC} + F_{CSTCNPDL1} - F_{CNPDL1CPDL1} - F_{TKCNPDL1} - F_{DCNPDL1}$$

$$\frac{dCPDL1}{dt} = F_{RESCPDL1}F_{TEXC}F_{CAFC} + F_{CSTCPDL1} + F_{CNPDL1CPDL1} - F_{TKNPDCPDL1} - F_{DCPDL1}$$

$$\frac{dCRNPDL1}{dt} = F_{RESCRNPDL1}F_{TEXCR}F_{CAFCR} + F_{CSNTCRNPDL1} - F_{TKCRNPDL1} - F_{CRNPDL1CRPDL1} - F_{DCRNPDL1}$$

$$\frac{dCRPDL1}{dt} = F_{RESCRPDL1}F_{TEXCR}F_{CAFCR} + F_{CSNTCRPDL1} + F_{CRNPDL1CRPDL1} - F_{TKCRPDL1} - F_{DCRPDL1}$$

$$\frac{dRES}{dt} = K_{RIN}(Y_{RESM} - RES) - K_{RESD}RES$$

$$\frac{dTKPD1}{dt} = F_{ProTKPD1}F_{THTKPD1}F_{IL2TK} - F_{TKPD1TKNPD1} - F_{TKPD1TEX} - F_{DTKPD1}$$

$$\frac{dTKNPD1}{dt} = F_{ProTKNPD1}F_{THTKNPD1}F_{IL2TK} + F_{TKPD1TKNPD1} - F_{DTKNPD1}$$

$$\frac{dTH}{dt} = F_{ProTH}F_{CANTH}F_{TREGTH} - F_{DTH}$$

$$\frac{dTREG}{dt} = F_{ProTREG}F_{CAFTREG} - F_{DTREG}$$

$$\frac{dTEX}{dt} = F_{ProTEX} + F_{TKPD1TEX} - F_{DTEX}$$

$$\frac{dFWT}{dt} = F_{ProFWT} + F_{CAFFWT} - F_{FWTCAF} - F_{DFWT}$$

$$\frac{dCAF}{dt} = F_{ProCAF}F_{M2CAF}F_{CANCAF}F_{OPNCAF} + F_{FWTCAF} - F_{CAFFWT} - F_{DCAF}$$

$$\frac{dMACM1}{dt} = F_{ProMACM1}F_{CANMACM1} + F_{M2M1} - F_{M1M2} - F_{DMACM1}$$

$$\frac{dMACM2}{dt} = F_{ProMACM2}F_{CAFMACM2} + F_{M1M2} - F_{M2M1} - F_{DMACM2}$$

$$\frac{dIL2}{dt} = F_{TKIL2} - F_{DIL2} + C_{IL2}$$

$$\frac{dLIF}{dt} = F_{CANLIF} + F_{CAFLIF} - C_{LIFKO}LIF$$

$$\frac{dIFNG}{dt} = F_{TKIFNG}F_{OPNIFNG} - F_{DIFNG}$$

$$\frac{dIL8}{dt} = F_{CANIL8} + F_{CAFIL8} + F_{M2IL8} - F_{DIL8} - C_{IL8KO}IL8$$

$$\frac{dLAC}{dt} = F_{CANLAC} + F_{MACLAC} - F_{DLAC} - C_{LIFKO}LAC$$

$$\frac{dIL10}{dt} = F_{TKIL10} - F_{DIL10}$$

$$\frac{dICAM1}{dt} = F_{TKICAM1} - F_{DICAM1}$$

$$\frac{dOPN}{dt} = (F_{CANOPN} + F_{CAFOPN})F_{IRF8OPN} - F_{DOPN} - C_{OPNKO}OPN$$

$$\frac{dIRF8}{dt} = F_{MACM1IRF8} - F_{DIRF8}$$
