# Supplementary material for: Tumor microenvironment governs the prognostic landscape of immunotherapy for head and neck squamous cell carcinoma: A computational model-guided analysis
Source: PLoS Comput Biol. 2025 Jun 3;21(6):e1013127. doi: 10.1371/journal.pcbi.1013127 (PMC12162103; doi:10.1371/journal.pcbi.1013127)
Supplement: S3 Text — (PDF) [file pcbi.1013127.s003.pdf]

S3 Text: Parameters varied for Figure 2

Parameter	Description	Range
K_{TumTex}	Exhausted T cell-driven proliferation of tumor cell states	0-2000
K_{TumCAF}	CAF-driven proliferation of Tumor cell states	0-2000
K_{TumTK}	Killer T cell driven elimination of tumor cell states	1500-2000
K_{TKPD1}	Natural proliferation of Killer T cells	0-2000
$K_{TKPDTEX}$	Exhaustion rate of Killer T cells	0-2000
K_{TKTH}	Helper-driven proliferation rate of killer T cells	0-2000
K_{TKIL2}	IL-2-driven proliferation rate of killer T cells	0-2000
K_{TKD}	Death rate of killer T cells	0-2000
K_{TH}	Natural proliferation of Killer T cells	0-2000
K_{THTum}	Tumor cell-driven proliferation of helper T cells	0-2000
K_{THD}	Death rate of helper T cells	0-2000
K_{TReg}	Natural proliferation rate of regulatory T cells	0-2000
$K_{TRegCAF}$	CAF-driven proliferation of regulatory T cells	0-2000
K_{TRegD}	Death rate of regulatory T cells	0-2000
K_{TEX}	Natural proliferation of exhausted T cells	0-2000

$\boldsymbol{K_{TEXD}}$	Death rate of exhausted T cells	0-2000
$\boldsymbol{K_{CAF}}$	Natural Proliferation of CAF	0-2000
$\boldsymbol{K_{CAFTum}}$	Tumor cells-driven proliferation of CAF	0-2000
$\boldsymbol{K_{CAFOPN}}$	OPN cells-driven proliferation of CAF	0-2000
$\boldsymbol{K_{FWTCAF}}$	Conversion from wild-type fibroblast to CAF	0-2000
$\boldsymbol{K_{CAFM2}}$	M2-macrophage driven growth of CAF	0-2000
$\boldsymbol{K_{CAFD}}$	Death rate of CAF	0-2000
$\boldsymbol{\alpha}$	Fraction of CAF engaged in collagen decomposition and physical barriers	[0, 0.02,0.2]
