# Supplementary material for: Tumor microenvironment governs the prognostic landscape of immunotherapy for head and neck squamous cell carcinoma: A computational model-guided analysis
Source: PLoS Comput Biol. 2025 Jun 3;21(6):e1013127. doi: 10.1371/journal.pcbi.1013127 (PMC12162103; doi:10.1371/journal.pcbi.1013127)
Supplement: S4 Text — (PDF) [file pcbi.1013127.s004.pdf]

S4 Text: Parameters fixed for Figure 2

Parameter	Description	Value	Supporting reference
$K_{ResCST}, K_{ResCSNT}$	Resource-driven proliferation of stem tumor cells (both immune accessible and inaccessible)	10	Halkola *et al* (24)
$K_{ResCPDL1}, K_{ResCRPDL1}$	Resource-driven proliferation of PDL1+ stem tumor cells (both immune accessible and inaccessible)	10	Halkola *et al* (24)
$K_{ResCNPDL1}, K_{ResCRNPDL1}$	Resource-driven proliferation of PDL1- stem tumor cells (both immune accessible and inaccessible)	12	Halkola *et al* (24)
α_C	Spatial competition across tumor cell states	0.005	Assumed (Low spatial competition)
$Y_{CST}, Y_{CNPDL1}, Y_{CPDL1}$	Carrying capacity for tumor cell states	10000, 10000, 10000	Mayer *et al.* (94)
$K_{CSTCNPDL1}$	Conversion from stem to PDL1- tumor cells	10	Assumed
$K_{CSTCPDL1}$	Conversion from stem to PDL1- tumor cells	12	Assumed

K_{LACTK}	Lactate-based inhibition of killer T cell activity	0.05	Assumed
K_{CSTD}	Natural death rate of tumor stem cells	0.0005	Assumed
$K_{IFNGCSTD}$	IFNG-driven death of tumor stem cells	1	Assumed
K_{M1CSTD}	M1 macrophage-driven death of tumor stem cells	1	Assumed
$K_{CNPDL1CPDL1}$	IFNG-driven conversion of the PDL1- tumor cells to PDL1+ tumor cells.	20	Assumed
$K_{CNPDL1D}$	Death rate of PDL1- tumor cells	8	Halkola *et al* (24) (Ratio between apoptosis of tumor cells and killer T cell-driven death)
K_{CPDL1D}	Death rate of PDL1+ tumor cells	8	Halkola *et al* (24) (Ratio between apoptosis of tumor cells and T cel-driven death)
δ	Skin depth associated with the killer T cells penetration of CAF-barrier	0.0001	Assumed

K_{CAFB}	Barrier formation rate by CAF	0.001	Assumed
Y_{TKM}	Total carrying capacity of Killer T cells	5000	Mayer *et al.* (94)
Y_{THM}	Total carrying capacity of helper T cells	5000	Mayer *et al.* (94)
Y_{TRegM}	Total carrying capacity of regulatory T cells	5000	Assumed
K_{TKNPD}	Proliferation rate of PD1- killer T cells	60	Halkola *et al* (24)
$K_{TKPDNPD1}$	Anti-PD1-driven conversion from PD1+ killer T cells	1500	Halkola *et al* (24)
Y_{RM}	Constant resource supply rate	1- Fixed	Halkola *et al* (24)
K_{ResD}	Resource degradation rate	8	Halkola *et al* (24)
K_{THTreg}	Regulatory T cell-driven inhibition of Helper T cells.	0.01	Halkola *et al* (24)
Y_{FM}	Total carrying capacity of the fibroblasts	5000	Mayer *et al.* (94)
K_{FWT}	Natural proliferation rate of wild type fibroblasts	50	Mayer et al. (94). High self-proliferation of fibroblasts compared to macrophages.

α_{LIF}	Fraction of LIF available for conversion to CAF	0.0005	Assumed
K_{CAFFWT}	Reverse conversion from CAF to wild type fibroblasts	9	Assumed
K_{FWTD}	Death rate of wild type fibroblasts	10	Assumed
Y_{MM}	Total carrying capacity of fibroblasts	5000	Mayer *et al.* (94)
K_{M1}	Natural proliferation of M1 macrophage	15	Assumed
K_{M2}	Natural proliferation of M1 macrophage	5	Assumed
K_{TumM1}	Proliferation of M1 macrophage via antigen sensing	10	Assumed
K_{M1M2}	IL-10-driven conversion from M1 to M2 macrophage	30	Assumed
α_{IL-10}	Fraction of IL-10 present in the vicinity of M1 macrophage	0.005	Assumed
K_{M2M1}	Conversion from M2 to M1 macrophage	3	Assumed
K_{M2CAF}	CAF-driven proliferation of M2 macrophage	120	Mayer *et al.* (94) (Highest ligand receptor score)
K_{M1D}	Death rate of M1 macrophage	10	Assumed
K_{M2D}	Death rate of M2 macrophage	10	Assumed

K_{TKIL2}	IL-2 secretion by killer T cells	5	Assumed
K_{IL2D}	Degradation rate of IL-2	6	Assumed
K_{TIFNG}	IFNG secretion by Killer T cell	20	Assumed
$K_{IFNGOPN}$	OPN-driven inhibition of IFNG	0.01	Assumed
K_{IFNGD}	Degradation rate of IFNG	5	Assumed
K_{IL10TK}	IL-10 secretion by killer T cells	5	Assumed
$K_{IL10TK}D$	IL-10 Degradation by killer T cells	5	Assumed
K_{TumOPN}	OPN secretion by tumor cells	3	Assumed
K_{CAFOPN}	OPN secretion by CAF	5	Mayer *et al.* (94) (Strong self loop of CAF)
$K_{OPNIRF8}$	IRF8-driven inhibition of OPN	0.05	Assumed
K_{OPND}	Degradation rate of OPN	4	Assumed
K_{TumLIF}	LIF secretion by tumor cells	6	Assumed
K_{CAFLIF}	LIF secretion by CAF	0.05	Assumed
K_{LIFD}	Degradation rate of LIF	8	Assumed
K_{TumIL8}	IL-8 secretion by tumor cells	2	Assumed
K_{CAFIL8}	IL-8 secretion by CAF	2	Assumed
K_{M2IL8}	IL-8 secretion by M2 macrophage	15	Assumed

K_{IL8D}	Degradation rate of IL-8	5	Assumed
K_{M1IRF8}	IRF8 secretion by M1 macrophage	2	Assumed
K_{IRF8D}	Degradation rate of IRF8	2	Assumed
K_{M2LAC}	Lactate secretion by M2 macrophage	2	Assumed
K_{TumLAC}	Lactate secretion by tumor cells	0.2	Assumed
K_{LACD}	Degradation rate of Lactate	4	Assumed
