# Supplementary material for: Tumor microenvironment governs the prognostic landscape of immunotherapy for head and neck squamous cell carcinoma: A computational model-guided analysis
Source: PLoS Comput Biol. 2025 Jun 3;21(6):e1013127. doi: 10.1371/journal.pcbi.1013127 (PMC12162103; doi:10.1371/journal.pcbi.1013127)
Supplement: S5 Text — (PDF) [file pcbi.1013127.s005.pdf]

S5 Text: Abbreviations

Res = Resource concentration

C_0 subtypes:
CST = Killer T cell-exposed Tumor stem cells
CSNT = Non-Killer T cell-exposed Tumor stem cells

C- subtypes:
CNPDL1 = Tumor cells exposed to Killer T cells without PDL-1
CRNPDL1 = Tumor cells hidden from Killer T cells without PDL-1

C+ subtypes:
CPDL1 = Tumor cells exposed to Killer T cells with PDL-1
CRPDL1 = Tumor cells hidden from Killer T cells with PDL-1

T cell subtypes:
TKPD1 = Killer T cells with PD-1 (TK+)
TKNPD1 = Killer T cells without PD-1 (TK-)
TH = Helper T cells
TREG = Regulatory T cells
TEX = Exhausted T cells

Fibroblast subtypes:
FWT = Wild type fibroblasts (F_WT)
CAF = Invasive cancer associated fibroblasts

Macrophage subtypes:
MACM1 = M1 phase macrophage
MACM2 = M2 phase macrophage

Molecular species:
IL2 = Interleukin 2
LIF = Leukemia inhibitory factor
IFNG = Interferon gamma
IL8 = Interleukin 8
LAC = Lactate
IL10 = Interleukin-10
OPN = Osteopontin
IRF8 = Interferon regulatory factor 8
