# Supplementary material for: Tumor microenvironment governs the prognostic landscape of immunotherapy for head and neck squamous cell carcinoma: A computational model-guided analysis
Source: PLoS Comput Biol. 2025 Jun 3;21(6):e1013127. doi: 10.1371/journal.pcbi.1013127 (PMC12162103; doi:10.1371/journal.pcbi.1013127)
Supplement: S6 Text — (PDF) [file pcbi.1013127.s006.pdf]

S6 Text: Self-assessment of adherence to the Ten Simple Rules of Credible Practice in Modeling and Simulation in Healthcare

The current self-assessment of the manuscript titled, ‘Tumor microenvironment governs the prognostic landscape of immunotherapy for head and neck squamous cell carcinoma: A computational model-guided analysis.’ is in accordance with Erdemir et al. (2020). The rubric can be accessed at: https://www.imagwiki.nibib.nih.gov/content/10-simple-rules-conformance-rubric

Date of self-assessment: August 29, 2024
Date of revised self-assessment: April 3,2025

Model files and documentation: Provided in the supplementary text.

Summary of revision: Based on the reviewer’s suggestion, we made the following changes

1. Added a python equivalent for our model that reproduces all the results of the manuscript.

2. Commented both the MATLAB and python files extensively for better understanding.

Rule 1: Define context clearly: Develop and document the subject, purpose, and intended use(s) of the model or simulation.

Current Conformance Level: Comprehensive

Model Context: Cell-state-specific mechanistic model of the tumor microenvironment (TME) for head and neck squamous cell carcinoma (HNSCC) in the presence (absence) of Immune checkpoint inhibitor treatment (ICI).

Primary goal of the model/tool/database: The primary goal of the modeling exercise was to leverage the TME-wide mechanistic models to explain (a) the existence of distinct compositional possibilities of the HNSCC TME and (b) how these compositional possibilities play a governing role in determining the response to ICI therapy. Additionally, the proposed model predicts the potential targets and biomarkers towards an improved ICI response.

Biological Domain of the Model: Cellular state

Structures of the Model: Tumor microenvironment

Spatial Scales Included in the Model: N/A (Assumes spatial homogeneity)

Time Scales Included in the Model: Week-Month

<u>Rule 2: Use contextually appropriate data:</u> Employ relevant and traceable information in the development or operation of a model or simulation.

Current Conformance Level: Adequate

Data for building the model	Published?	Private?	How is credibility checked?	Current Conformance Level
in vitro (primary cells cell, lines, etc.)	N/A	N/A	N/A	N/A
ex vivo (excised tissues)	N/A	N/A	N/A	N/A
in vivo pre-clinical (lower-level organism or small animal)	N/A	N/A	N/A	N/A
in vivo pre-clinical (large animal)	N/A	N/A	N/A	N/A
Human subjects/clinical	Yes	No	The source data is qualitative and the related clinical protocols have been published in peer-reviewed journals	Adequate

Data for validating the model	Published?	Private?	How is credibility checked?	Current Conformance Level
in vitro (primary cells cell, lines, etc.)	N/A	N/A	N/A	N/A
ex vivo (excised tissues)	N/A	N/A	N/A	N/A
in vivo pre-clinical (lower-level organism or small animal)	N/A	N/A	N/A	N/A
in vivo pre-clinical (large animal)	N/A	N/A	N/A	N/A

Human subjects/clinical	Yes	No	The source data is qualitative and the clinical protocols have been published in peer-reviewed journals	Adequate

Rule 3: Evaluate within context: Perform verification, validation, uncertainty quantification, and sensitivity analysis of the model or simulation with respect to the reality of interest and intended use(s) of the model or simulation.

Current Conformance Level: Extensive

	Who Does It?	When does it happen?	How is it done?	Current Conformance Level
**Verification**	Developer	During development	Comparison of model output with the experimental and clinical observations	Extensive
**Validation**	Lab Member	During development	model was used to reproduce simulations and figures	Extensive
**Uncertainty Quantification**	User performs uncertainty quantification	Can be performed every time the model is run for a new scenario	User discretion	Adequate
**Sensitivity Analysis**	User performs sensitivity analysis on influential parameters.	Can be performed after every new simulation	User discretion	Adequate

<u>Rule 4: List limitations explicitly:</u> Provide restrictions, constraints, or qualifications for or on the use of the model or simulation for consideration by the users or customers of a model or simulation.

Current Conformance Level: Comprehensive

Disclaimer statement (explain key limitations)	Who needs to know about this disclaimer?	How is this disclaimer shared with that audience?	Current Conformance Level
Models are limited by the spatial homogeneity approximation	Users	Stated in the main text	Comprehensive
Model does not capture metastasis and the associated transition and other necessary interactions	Users	Stated in the main text	Comprehensive
The conclusion drawn from this model are conditioned on the particular modeling rules specified in the main text	Users	Stated in the main text	Comprehensive

<u>Rule 5: Use version control:</u> Implement a system to trace the time history of modeling and simulation activities including delineation of each contributors' efforts.

Current Conformance Level: Extensive

	**Naming Conventions?**	**Repository?**	**Code Review?**
**individual modeler**	N/A	Github	Yes
**within the lab**	Yes	Yes	Yes
**collaborators**	N/A	Github	Yes

<u>Rule 6: Document appropriately:</u> Maintain up-to-date informative records of all modeling and simulation activities, including simulation code, model mark-up, scope and intended use of modeling and simulation activities, as well as users' and developers' guides.

Current Conformance Level: Extensive

	**Current Conformance Level**
**Code Commented?**	Extensive: Commented the codes in every important line of the code for better interpretability.
**Scope and intended use described?**	Extensive: Described in the beginning of the each section of the code and in the main text.
**User's Guide**	Extensive: described in the main text and supplemental files
**Developer's Guide?**	Adequate: Described in the main text and the supplementary files.

Rule 7: Disseminate broadly: Share all components of modeling and simulation activities, including

simulation software, models, simulation scenarios and results.

Current Conformance Level: Extensive

**Target Audience(s):**	**"Inner Circle"**	**Scientific Community**
**Simulations**	Shared with the lab members for replication	The co-authors of this work presented this in the form of posters in FOSBE2024, VPH2024
**Models**	Shared with the lab members for replication	The co-authors of this work presented this in the form of posters in FOSBE2024, VPH2024
**Software**	MATLAB is well-known software in the lab of the first author.	MATLAB is well-known software in the mathematical modeling community.
**Results**	Presented at lab members	MATLAB is well-known software in the lab of the first author.
**Implication of Results**	Presented at lab members	MATLAB is well-known software in the lab of the first author.

Rule 8: Get independent reviews: Have the modeling and simulation activity reviewed by nonpartisan third-party users and developers.

Current Conformance Level: Extensive

**Reviewer(s) name and affiliation**	**Alexandra Manchel (Thomas Jefferson University)**
When was the review performed?	First Version: August 15, 2024<br>Second version: April 3, 2024
How was review performed and outcomes of the review?	A member of the research group, not involved in the present study and does not conduct research in tumor microenvironment modeling, performed the review. Model scripts were cross-checked for consistency. Simulation results and figures were replicated using the files provided on GitHub.

**Reviewer(s) name and affiliation**	**Dr. Prem Jagadeesan (Purdue university)**
When was the review performed?	August 29, 2024
How was review performed and outcomes of the review?	A member of the research group, not involved in the present study and does not conduct research in tumor microenvironment modeling, independently produced the Figure 3 of the manuscript from the model equations, parameters, and initial conditions provided on GitHub.

<u>Rule 9: Test competing implementations</u>: Use contrasting modeling and simulation implementation strategies to check the conclusions of different strategies against each other.

Current Conformance Level: Adequate

	**Yes or No (briefly summarize)**
**Were competing implementations tested?**	Competing implementations were conceptualized by all the authors and tested by the first author of this manuscript.
**Did this lead to model refinementor improvement?**	Yes, the completing implementations led to modifications and refinements of the model.

<u>Rule 10: Conform to standards:</u> Adopt and promote generally applicable and discipline specific operating procedures, guidelines, and regulations accepted as best practices.

Current Conformance Level: Adequate

	**Yes or No (briefly summarize)**
**Are there operating procedures, guidelines, or standards for this type of multiscale modeling?**	Yes, the existing modeling approaches for tumor microenvironment employs continuous-time mathematical models which can vary between deterministic and stochastic settings.
**How do your modeling efforts conform?**	The proposed model adopts a continuous time modeling framework in a deterministic setting. Therefore, the modeling framework conforms to the existing conventions in the modeling literature.

References:

1. Erdemir, A.; Mulugeta, L.; Ku, J.P.; Drach, A.; Horner, M.; Morrison, T.M.; Peng, G.C.Y.; Vadigepalli, R.; Lytton, W.W.; Myers, J.G. Credible Practice of Modeling and Simulation in Healthcare: Ten Rules from a Multidisciplinary Perspective. *J. Transl. Med.* **2020**, *18*, 369, doi:10.1186/s12967-020-02540-4.
