# Supplementary figures and images for: Tumor microenvironment governs the prognostic landscape of immunotherapy for head and neck squamous cell carcinoma: A computational model-guided analysis
Source: PLoS Comput Biol. 2025 Jun 3;21(6):e1013127. doi: 10.1371/journal.pcbi.1013127 (PMC12162103; doi:10.1371/journal.pcbi.1013127)

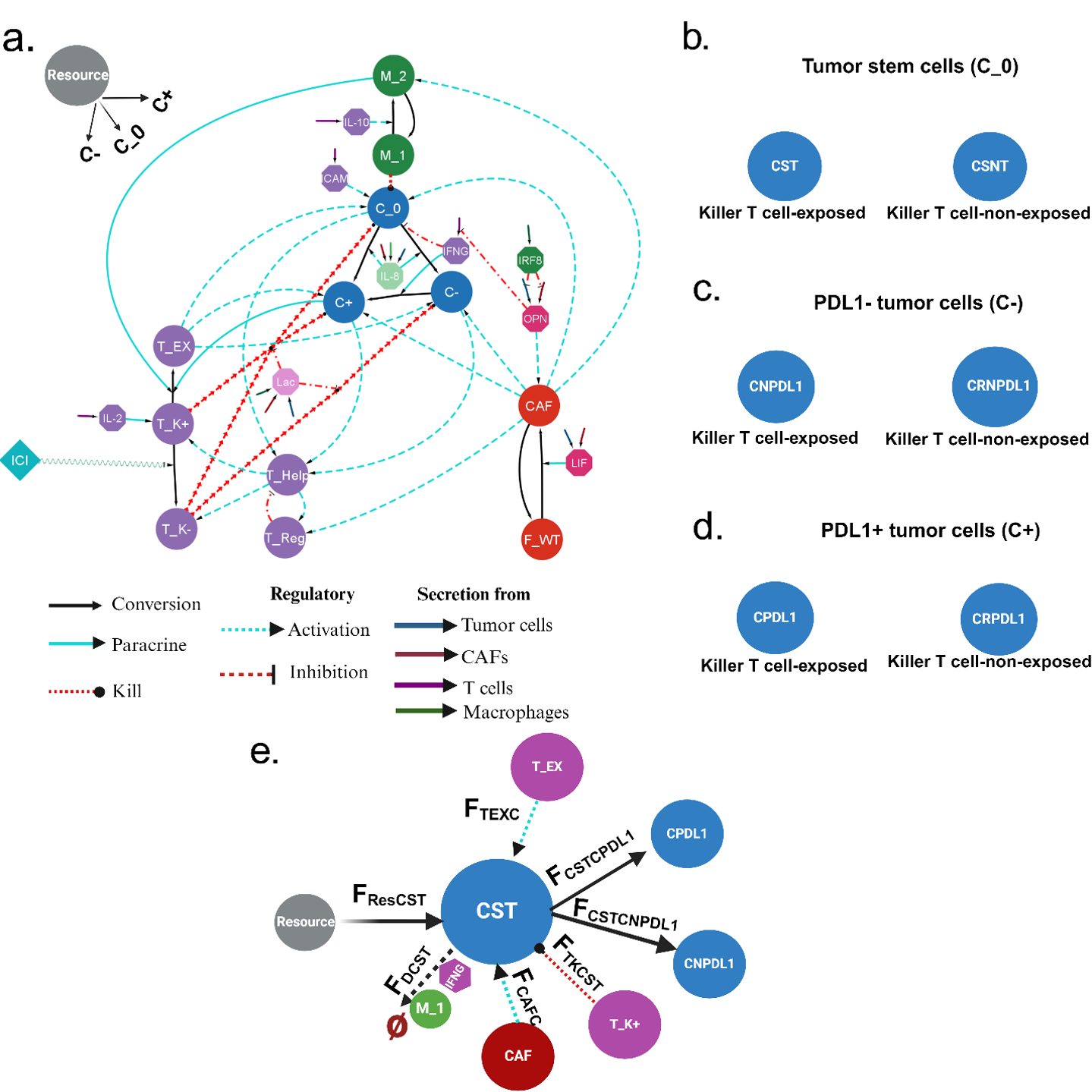

Supplement: S1 Fig — (a) The nodes are either the cell states or the molecular species, whereas the edges represent diverse forms of interactions. The acronyms C_0, C + , and C– refer to stem, PDL1+ (programed death ligand1), and PDL1– tumor cells, respectively. T_K + , T_K-, T_Help, T_Reg, and T_EX stands for PD1+ (programmed death 1), PD1- killer T cells, Helper T cells, Regulatory T cells, and Exhausted T cells, respectively. M_1 and M_2 refer to macrophages of M1 and M2 phase, respectively. Further, F_WT and CAF correspond to wild type and invasive cancer associated fibroblasts, respectively. The acronyms IL-2, IL-8, IL-10 LIF, IFNG, IRF8, OPN, ICAM1, and Lac denote Interleukin 2, Interleukin 8, Interleukin 10, Leukemia Inhibitory Factor, Interferon Gamma, Interferon Regulatory Factor 8, Osteopontin, Intercellular Adhesion Molecule 1, and Lactate, respectively. All the cell states are assumed to be capable of self-proliferation and natural death. Therefore, the self-loops are not shown for brevity. (b-d) Each tumor cell state is subdivided depending on the accessibility from the Killer T cells. The Killer T cell-exposed tumor cells are exposed to immune response whereas the Killer T cell-non-exposed tumor cells are protected by the CAF-derived barrier from immune onslaught. (e) Flux-structure mapping for the killer T-cell-expose tumor stem cells. (TIF) [file pcbi.1013127.s007.tif]

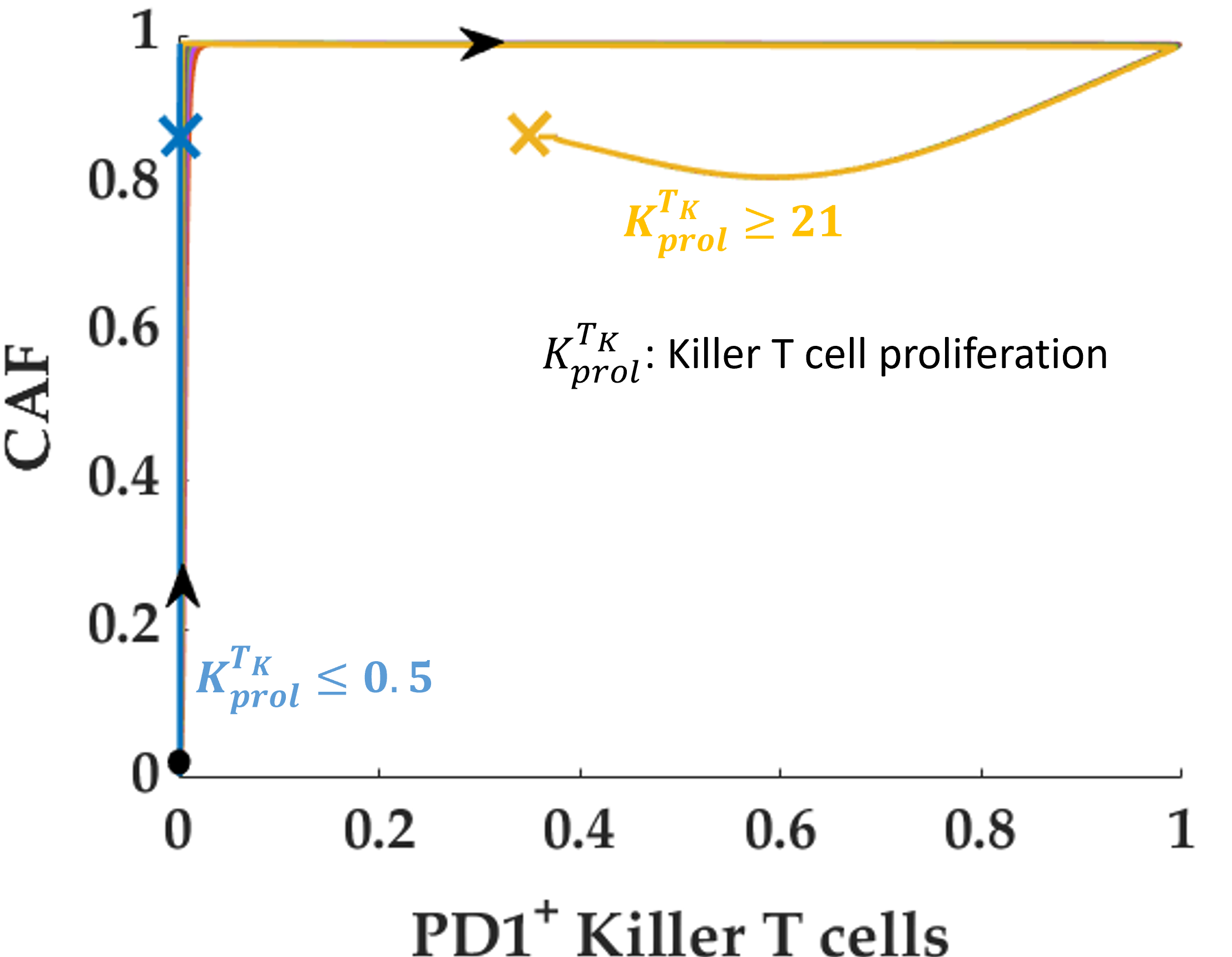

Supplement: S2 Fig — The proliferation rate governs the pre-ICI population of killer T cells. Below a critical proliferation rate the HNSCC TME model settles in an immune-desert region. Whereas, in both the scenarios (immune-desert and immune-rich), the CAF population remains unaltered indicating a relative independence from the T cell population. (TIF) [file pcbi.1013127.s008.tif]

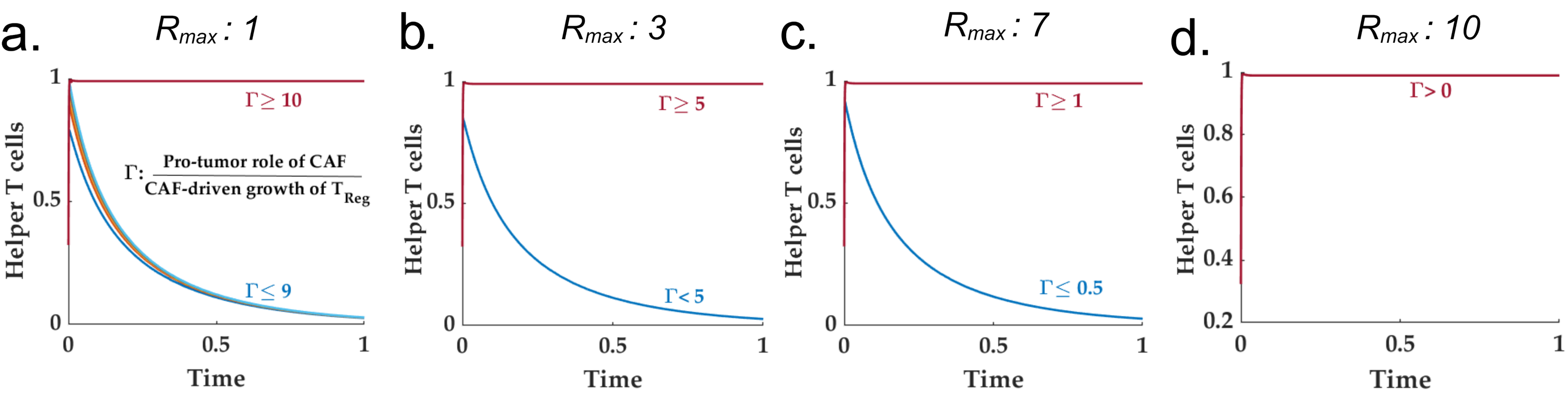

Supplement: S3 Fig — (a-d) The pro-tumor role of CAF leads to significant pre-ICI, PDL1- tumor cell population. Therefore, beyond a threshold value of the pro-tumor role of CAF (compared to the CAF-driven growth of regulatory T cells), the final helper T cell population remains high. On the other hand, for moderate to low CAF-tumor interaction, the PDL1- tumor cells remain low due to the presence of cytotoxic killer T cells. Therefore, despite an initial increase, the helper T cells settle to a very low value (almost zero). Further, the threshold value of CAF-tumor interaction is dependent on the maximum resource intake in a competitive setting. (TIF) [file pcbi.1013127.s009.tif]

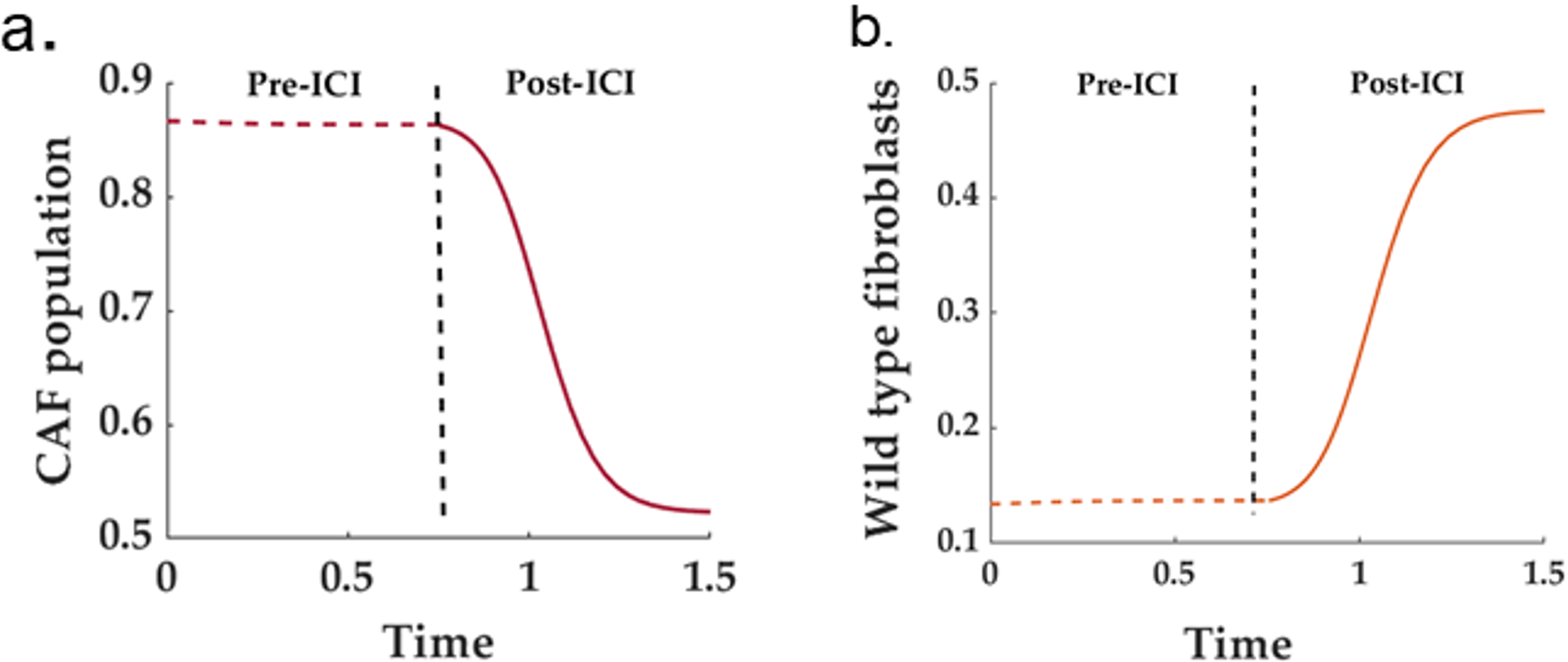

Supplement: S4 Fig — (a) The CAF population exhibits a steep increasing tendency owing to multiple paracrine interaction with the tumor cells and tumor associated macrophages. However, the ICI intervention in an immune rich scenario reduces the tumor cells. Further, the reduction in tumor cells-secreted LIF reduces the transition flux from wild type to cancer-associated fibroblasts. Therefore, overall CAF population undergoes a significant reduction during the ICI therapy. (b) The wild-type fibroblast population, due to significant reduction in the transition flux towards CAF, increases during an ICI-based therapy in immune rich scenario. (TIF) [file pcbi.1013127.s010.tif]

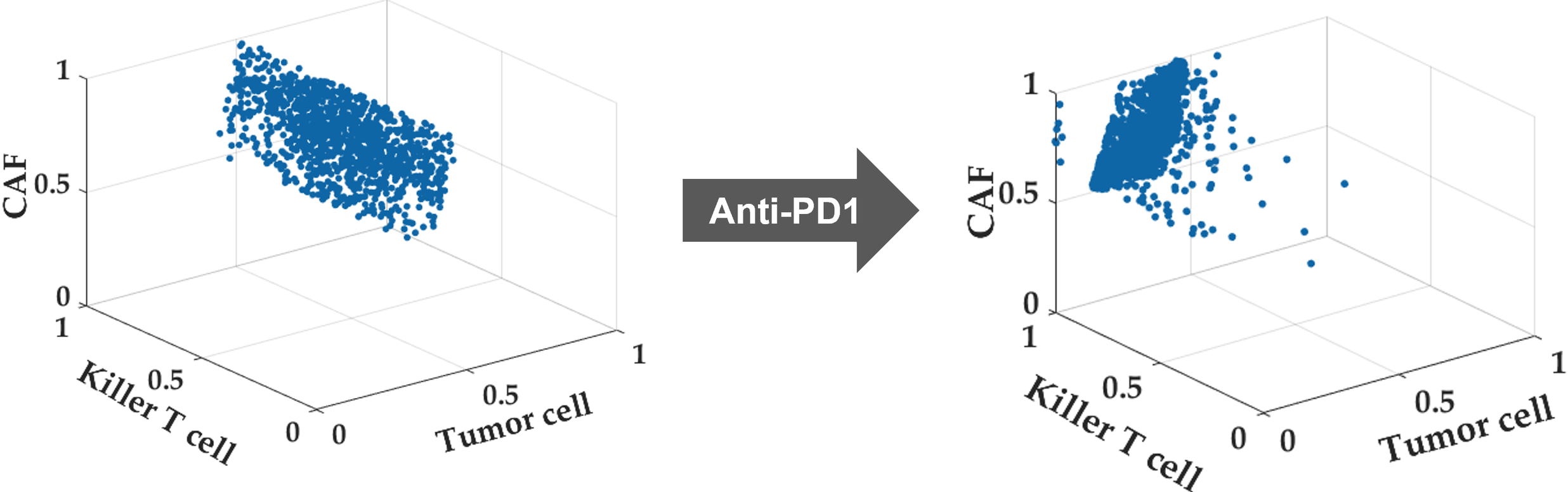

Supplement: S5 Fig — (a) Possible tumor cell, Killer T cell, and CAF populations within the subset of the parameters (selected for Figure 2) corresponding to the immune-dominated subtype (Group-4). The application of anti-PD1 results in the significant reduction (complete removal in a few scenarios) of the tumor cells, CAF population. On the other hand, the anti-PD1significantly increases the resident killer T population in the TME. (TIF) [file pcbi.1013127.s011.tif]

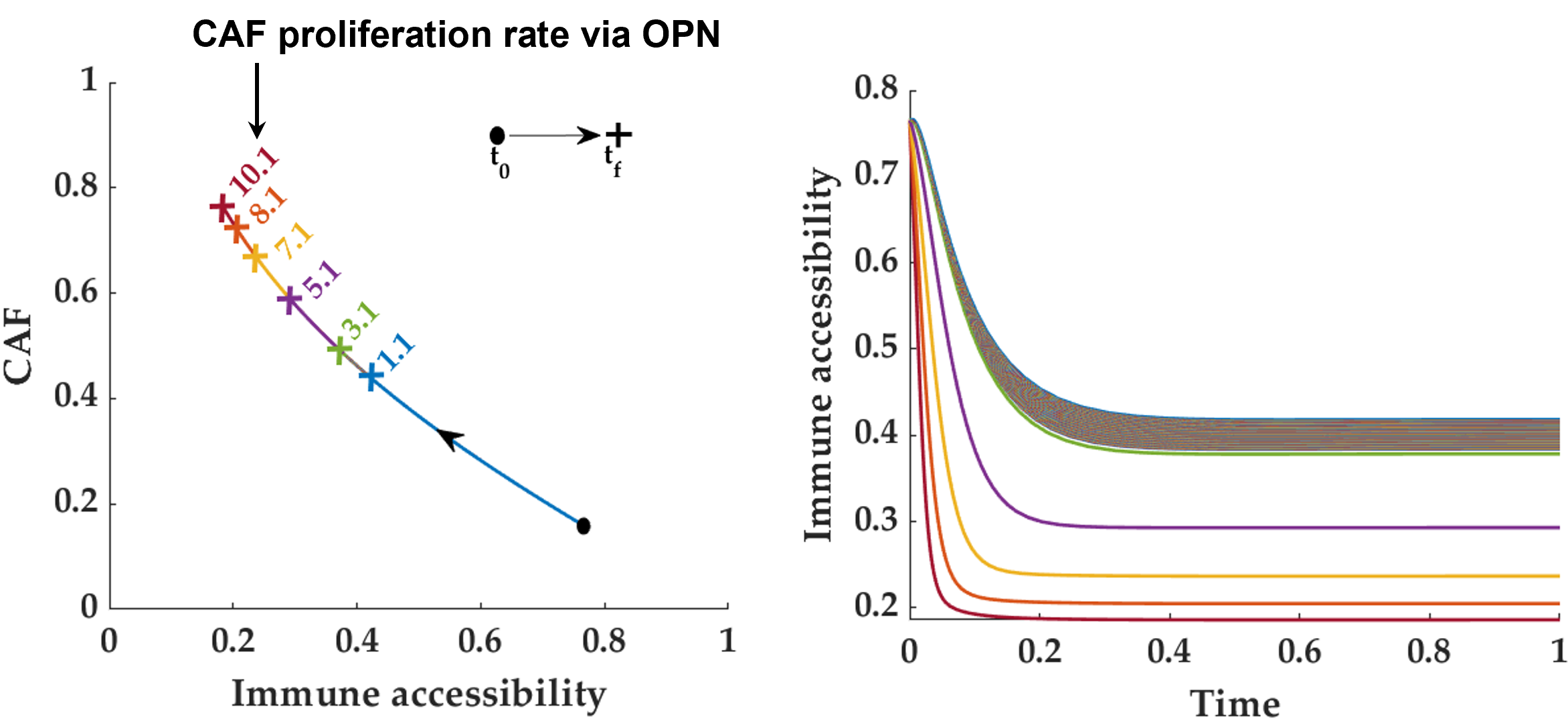

Supplement: S6 Fig — (a) Demonstrates the phase-space between the immune accessibility and CAF for different CAF proliferation rate. (b) The time profile for immune accessibility shows the existence of a threshold time beyond which the immune accessibility deteriorates drastically. Further, this threshold time is dependent on the proliferation rate of CAF. This is due to the fact that a higher proliferation rate renders a faster CAF growth and due to the near-linear trajectory of CAF-immune accessibility trajectory, the immune accessibility adopts a faster time scale. (TIF) [file pcbi.1013127.s012.tif]

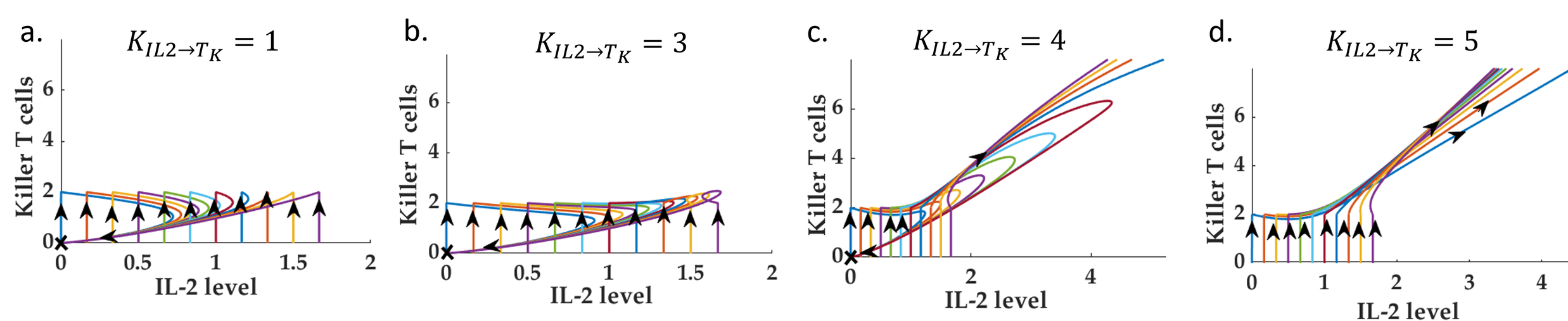

Supplement: S7 Fig — (a-d) Increasing levels of IL-2-induced killer T cell proliferation rate can drive the HNSCC immune-desert TME to an immune hot scenario. However, there exists a threshold IL-2-driven Killer T cell proliferation rate below which the immune-desert scenario can not be circumvented irrespective of the external IL-2 level. (TIF) [file pcbi.1013127.s013.tif]

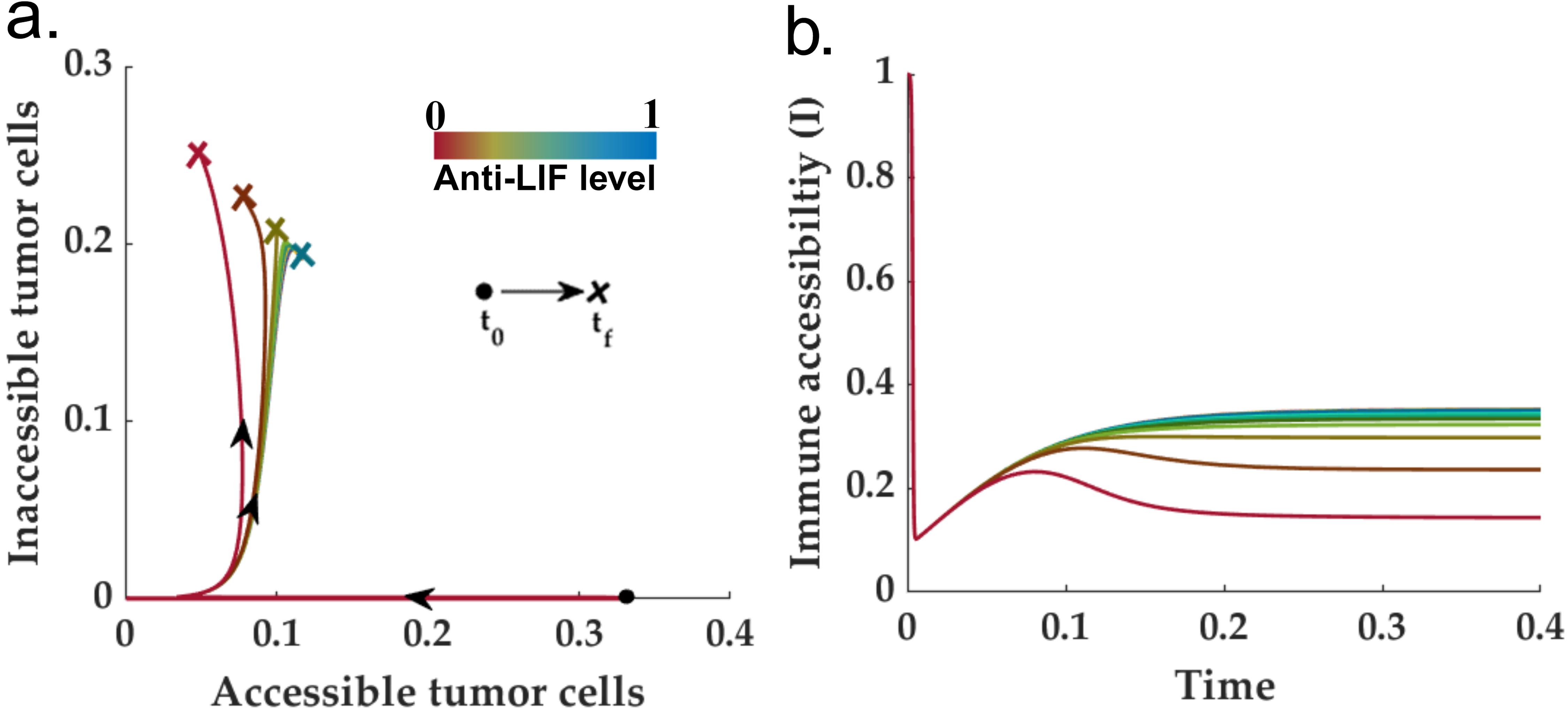

Supplement: S8 Fig — (a) Phase trajectory of different tumor cells vis-à-vis immune accessibility. The LIF knockout significantly reduces the pre-ICI inaccessible tumor cells. However, a complete (or near) complete elimination of inaccessible tumor cells is not possible with only LIF knockout. (b) Although a LIF knockout improves the immune accessibility it does not drive the TME system to an immune-dominated situation. (TIF) [file pcbi.1013127.s014.tif]

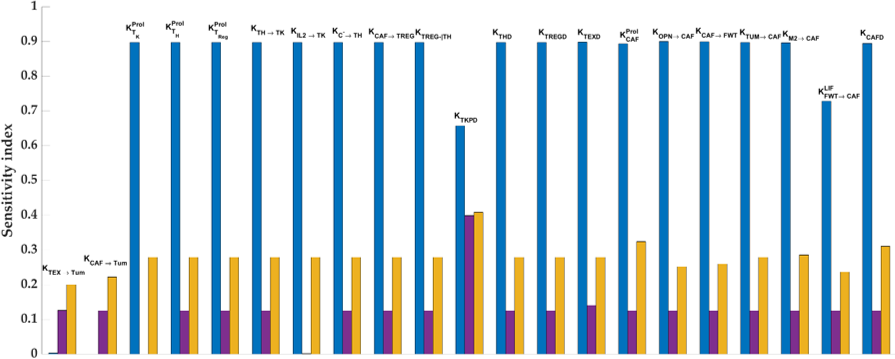

Supplement: S9 Fig — We chose all the parameters that exhibits an explicit bearing with the proliferation and death and conversion fluxes for Tumor cells (Blue), Killer T cells (Violet), and CAF (Yellow). (TIF) [file pcbi.1013127.s015.tif]
