# Supplementary material for: Tumor microenvironment governs the prognostic landscape of immunotherapy for head and neck squamous cell carcinoma: A computational model-guided analysis
Source: PLoS Comput Biol. 2025 Jun 3;21(6):e1013127. doi: 10.1371/journal.pcbi.1013127 (PMC12162103; doi:10.1371/journal.pcbi.1013127)
Supplement: Attachment — Submitted filename: Response to Review.pdf [file pcbi.1013127.s017.pdf]

Tumor microenvironment governs the prognostic landscape of immunotherapy for head and neck squamous cell carcinoma: A computational model-guided analysis

Reviewers' Comments:

Reviewer #1: The biology examined in the paper is not my area of expertise so I won't comment on the quality of the biological research. Instead I will make some, hopefully useful comments, on the modeling approach. The model is a straight forward, though non-trivial reaction/interaction based model, that can be described using differential equations. The authors chose to use MATLAB to distribute the model. What this means is that the model cannot be easily reused and requires a third-party to purchase a proprietary license for MATLAB which reduces access. The use pf Python or Julia would address access but not the reuse issue. In addition, there is now ample evidence (Bayesian estimation reveals that reproducible models in Systems Biology get more citations Sebastian Höpfl et al), that shows that models encoded in open standards are more cited and therefore have more impact that ones that are not. The authors do provide the MATLAB code which is quite dense on inspection so in principle the work can be repeated but no easily reproduced. It also means the model won't appear in any of the major model repositories, which is unfortunate. I am not expecting the authors to recode their model other than to suggest in the future they might use more open science based methodologies to publish their work. I also note their use the 10 rules of credible practice but only appear to conform to 3 of the rules. Admittedly this is better than most :)

Response: We thank the reviewer for reading through the manuscript and providing valuable suggestions and inputs. We would like to elaborate on the main themes of reviewer's comments.

Accessibility: We appreciate the reviewer's concern on the accessibility of the work. Therefore, we have appended the necessary python code for our model implementation. We have verified the python code output with the MATLAB implementation. The python version is publicly available on the GitHub repository along with the original MATLAB version in the Replication folder.

Repeatability and reproducibility: We have verified repeatability with one of the lab members of the Vadigepalli lab. Additionally, we have now extensively commented the MATLAB code for additional scaffolding to aid in the repeatability of the model. We demonstrate model reproducibility with a third party who generated the same results with the supplied parameter values and initial conditions.

Moreover, the reproduction folder in the GitHub repository contains the figure-wise necessary parameter values and initial conditions and the mathematical model can be built from the detailed flux equations provided in the 'HNSCC_model_equation.docx' file.

Ten simple rules: We would like to clarify that the modified version of the manuscript have the following features vis-à-vis the ten simple rules for modeling.

The modeling exercise defines the context clearly, states the model assumptions clearly, implements version control on GitHub. Further, we have commented on the code extensively to make it self-explanatory, and each code file uploaded on GitHub is accompanied with the parameter

values and initial conditions (and the globally applicable model equations in the context of this work) for the purpose of reproduction. The codes, model equations, and parameters are freely accessible to the user. We also had the model reviewed by Dr. Alexandra Manchel and Dr. Prem Jagadeesan who were not part of this study. We have also mentioned their current affiliations and contacts. We have also mentioned the competing scenarios tested and how they led to the refinements of the model in the supplementary information of the modified manuscript. Based on our knowledge, using continuous-time mathematical models (deterministic and stochastic) has been one of the widely used modelling strategies (along with the spatial, agent-based models) at the cellular level. As the reviewer correctly pointed out, the proposed model adopts a continuous-time, deterministic framework which does conform to the aforementioned continuous time modeling paradigm. However, we would also like mention that while a number of experimental results conform to all the major predictions of the model (Table 2 of the modified manuscript) evaluating the model predictions vis-a-vis quantitative prediction remains a future work.

Reviewer #2: In this manuscript, Bhattacharya et al model key cellular and molecular interactions within a tumor microenvironment to understand resistance to immune checkpoint inhibition (ICI) therapy. Overall, this is a comprehensive piece of work focused on profiling the composition of the tumor microenvironment. It also maps the response to different immune therapies based on cellular composition. The models are generated based on specific hypotheses and a breadth of scenarios are discussed in the manuscript. Overall, this is a strong and well-executed study. However, I have a few concerns:

Response: We thank the reviewer for the positive assessment of the manuscript.

1. The overall narrative is somewhat fragmented. The manuscript could benefit from some consolidation and a better-focused storyline.

Response: We appreciate the reviewer's concern. We have added a brief paragraph at the at the end of the introduction section (line numbers **160-165**) that lays out the overall outline of the manuscript. We have also explicitly stated the overall modeling goals in the beginning of the results section (line numbers **168-176** of the modified manuscript). Further, in order to improve the flow of the manuscript, we have appended few lines at the end of each subsection in the result section that links it to the results presented in the subsequent section.

2. The network in Fig. 2 is not described in sufficient detail in the text. A more comprehensive description of Fig. 2A should be added.

Response: We agree that figure 2 is one of the central results of the model. Therefore, considering the suggestion made by the reviewer, we dedicate an additional paragraph (line numbers **177-200** of the modified manuscript) that describes the figure panel 2(a) in the result section. Further, we have added a detailed description of the network construction in the materials and methods section (line numbers **811-834** of the modified manuscript).

3. It is difficult to tell the differences between Figs. 3e and 3f (with and without lactate). These differences should also be appropriately linked to corresponding conclusions.

Response: We thank the reviewer for pointing this out. We have now added a brief paragraph describing the difference between the Fig. 3(e) and Fig. 3(f) along the following lines (line numbers **336-347** of the modified manuscript). Additionally, we have modified both the figures to make it easy for the reader to differentiate between the Figs 3e and 3f.

The dotted line in Fig 3(e) shows that the ICI intervention does not yield any significant improvement in the overall trajectory of tumor development for immune desert TME caused by low killer T cell proliferation. On the contrary, the application of ICI does impact the tumor development trajectory in the scenario of exhaustion-driven immune desert TME (solid-colored lines in Fig. 3 (e)). Further, as seen from Fig. 3(f), the ICI-driven reduction of tumor cells can be further intensified with lactate knockout for exhaustion-driven immune desert scenario. However, the low-proliferation scenario remains nearly unimpacted by change in lactate levels. This inspires two primary conclusions that (a) the response of an immune-desert TME subtype to ICI is dependent on the desert mechanism and (b) the lactate-based combination strategies (along with ICI) to improve the prognosis for HNSCC patients with immune desert TME phenotype should be formulated based on desert mechanism.

4. The authors do a good job of describing their model assumptions. However, they should provide additional details regarding specific biological contexts where these assumptions hold vs where they don't.

Response: We have adopted the reviewer's suggestion and expanded the discussion section that includes the specific biological contexts that map the assumptions. We have also included the relevant contexts that violate our model assumptions thereby indicating the potentials for future interventions. We thank the reviewer for this important suggestion. Line numbers **787-795** in the modified manuscript reflect the suggested modifications.

5. There are some typos and incorrect figure calls. These should be corrected.

Response: We regret the errors. We have now thoroughly scanned through and modified the manuscript in line with the reviewer's observation.

Reviewer #3: Bhattacharya et al describe in their manuscript a model that governs the response to immunotherapy. The mathematical modeling part is impressive, but I believe that the current version of the manuscript lacks three major parts: 1. Detailed description of how the selection of parameters was done. 2. Description of how this model would be used in the clinic. 3. Any kind of validation that the proposed model actually works. Adding these three items would significantly improve the manuscript and its impact.

Response: We thank the reviewer for the valuable suggestions. We agree that the pertinent points raised by the reviewer have significantly improved the quality and impact of the manuscript. We also list out the pointwise clarification (and the actions taken) below.

1. Detailed description of how the selection of parameters was done. The current version lacks any kind of detailed description of why certain parameters were selected and, more importantly, why some obvious parameters are missing. For example, it is well established that B cells play an important role in immune response and this is not included. I'm also surprised that HPV status is not considered or mentioned. This may be all correct, but it does warrant some discussion. Would a non-relevant parameter still generate similar results as the selected ones? Were other parameters initially tested but did not work that well and were thus left out in the final version? Should ease of measurement be considered when selecting parameters?

Response: In the beginning of the Result section we spent a significant portion to discuss the rationale behind the construction of the TME network (line numbers **177-200** of the modified manuscript) to brief the rationale behind the construction of the model network). Further, we have also added a subsection under the 'Materials and Methods' section that describes the model components in sufficient details (line numbers **811-834** of the modified manuscript). We would like to point out that each edge in the proposed network (Fig. 2(a) of the revised manuscript) is supported by at least one experimental evidence published in the literature of HNSCC or similar solid tumors such as melanoma.

We agree with the reviewer that the human papillomavirus (HPV) signature remains an important factor in modulating the progress of HNSCC. The prognosis of HPV positive HNSCC patients are significantly better than HNSCC-negative patients owing to a strong immune infiltration program in HPV-positing HNSCC patients (Tosi *et al.* (2022)). The recent work by Puram et al. (2017) on the sc-RNA seq of 17 HNSCC patients across HPV signatures revealed that the overall cell types present in the HNSCC tumor microenvironment remains similar across HPV phenotypes. However, the compositions of different tumor and non-tumor cell types are different. To put these observations into the modeling context, we argue that the modeling scenarios with low immune cells or low immune accessibility typically refers to the HPV negative scenario.

We have rewritten the relevant results that connects to the HPV-signature specific observations for HNSCC (line numbers **273-278**, **588-593** of the modified manuscript). We have also included few lines in the discussion section along these lines (line numbers **777-784** in the Discussion section of the manuscript).

Although the current version of the model does not explicitly include B cells, it captures the functionality of B cells through an additional repressing edge from the M1 macrophage to the tumor cell states. Further, the exhaustion of PD1+ B cells pre-ICI scenario is similar to the T cell exhaustion

program in the presence of PDL1+ tumor cells states and M2 macrophages. Therefore, the overestimation of tumor cells due to the absence of mature B cells in the proposed model can be compensated by modulating the exhaustion rate of killer T cells and repression of tumor cells by M1 macrophages.

Line numbers **822-834** of the modified manuscript contains a brief description regarding the inclusion of B cells along the aforementioned lines.

Overall, we have attempted to construct an essential set of reaction atlas and minimal number of nodes that capture the well-known clinically observed features of the HNSCC TME. Therefore, while the set of nodes in the proposed network (Fig 2a of the modified manuscript) is not exhaustive, the simulation studies explored in the current version of the model agree with the major observations reported in the literature and make testable predictions regarding the identification of the target species for potential combination therapies along with anti-PD1 treatment to circumvent ICI resistance.

2. Description of how this model would be used in the clinic. From a practical point of view, how would the model translate to a CLIA-approved test? What measurements are needed to derive the required input variables for a patient? How would previous treatments, such as radiation and/or chemo change the results? Does the model require spatial transcriptomics data, or would some much more simple measurements work? This is important to know to be able to understand the clinical relevance of the manuscript.

Response: We appreciate the reviewer's exhortation to explain in detail the trajectory of how our modeling approaches can be evolved for eventual clinical use. We have added a new paragraph in the discussion section that addresses the reviewer's concern along the following lines:

The model requires the concentration profiles of the different cell states mentioned in the proposed network. This can be directly obtained through the measurements of the proportions of the cell counts for the cell types explored in the proposed network over multiple time points. Conversely, this can also be obtained through the dynamic single cell RNA sequencing of the HNSCC primary tumor. Using the tSNE-based clustering, it is possible to find out relative population of each cell states required for the construction of the model. Further, the plasma level concentration measurements of the molecular species (chemokines, cytokines, and lactate) are required for assessing the veracity of the model predictions.

Although the main focus of this model is immune checkpoint inhibition (ICI) therapy, the model can also be exploited to discuss the potential impact of chemotherapy along with checkpoint inhibition. Chemotherapy primarily modulates the natural proliferation rate of the tumor cells. Therefore, making the resource-driven proliferation rate as a function of drug dosage and appending chemotherapy-induced loss of immune cells in the existing model should address the question of the effect of combination (chemo and ICI) therapy.

We would also like to point out that the model is not meant for a direct clinical deployment. The primary purpose of the model in this study was to propose an integrated mechanistic framework for the purpose of mapping the existing knowledge about diverse cell-cell interactions in HNSCC to the clinical outcomes in the context of immune checkpoint inhibitor and additionally, identify potential

target species and biomarkers towards better prognosis. The next step towards building a personalized (clinically deployable) model requires construction of population-specific distribution of the key parameters in human cohorts. This is out of scope for the present work.

We have added a paragraph in the Discussion section (line numbers **761-772**) along the aforementioned lines.

3. Any kind of validation that the proposed model actually works. In the current version, we have no idea if the proposed model actually works. This makes all the discussion and questions asked in the Result section questionable. Basically, Figure 2 to Figure 10 all assume that the model is correct. It feels a bit redundant that so much space is given to interpret a model that the authors built themselves. Could any public dataset be used to show that the model works. There must be some spatial transcriptomics or single-cell dataset available.

Response: We appreciate the reviewer's concern. We start with adding a separate subsection in the 'Materials and methods' section that discuss all the experimental and clinical observations that validates the construction of the proposed TME network model for HNSCC.

Further, all the model predictions have been collated in the Table 2 of the modified manuscript wherein for every model prediction, we have placed the supporting evidences from the existing literature. We have also spent a few lines (line numbers **245-247**, **308-314**, **473-488**, and **588-593** of the modified manuscript) after each simulation case study that place the model predictions in the context of the existing literature.
